# Roles and Competencies of Doctors in Artificial Intelligence Implementation: Qualitative Analysis Through Physician Interviews

**DOI:** 10.2196/46020

**Published:** 2023-05-18

**Authors:** Masashi Tanaka, Shinji Matsumura, Seiji Bito

**Affiliations:** 1 Department of Clinical Epidemiology Tokyo Medical Center National Hospital Organization Tokyo Japan; 2 Department of Medical Ethics Tohoku University Graduate School Sendai Japan; 3 KARADA Internal Medicine Clinic Tokyo Japan; 4 Matsumura Clinic Tokyo Japan

**Keywords:** artificial intelligence, shared decision-making, competency, decision-making, qualitative research, patient-physician, medical field, AI services, AI technology

## Abstract

**Background:**

Artificial intelligence (AI) is a term used to describe the use of computers and technology to emulate human intelligence mechanisms. Although AI is known to affect health services, the impact of information provided by AI on the patient-physician relationship in actual practice is unclear.

**Objective:**

The purpose of this study is to investigate the effect of introducing AI functions into the medical field on the role of the physician or physician-patient relationship, as well as potential concerns in the AI era.

**Methods:**

We conducted focus group interviews in Tokyo’s suburbs with physicians recruited through snowball sampling. The interviews were conducted in accordance with the questions listed in the interview guide. A verbatim transcript recording of all interviews was qualitatively analyzed using content analysis by all authors. Similarly, extracted code was grouped into subcategories, categories, and then core categories. We continued interviewing, analyzing, and discussing until we reached data saturation. In addition, we shared the results with all interviewees and confirmed the content to ensure the credibility of the analysis results.

**Results:**

A total of 9 participants who belonged to various clinical departments in the 3 groups were interviewed. The same interviewers conducted the interview as the moderator each time. The average group interview time for the 3 groups was 102 minutes. Content saturation and theme development were achieved with the 3 groups. We identified three core categories: (1) functions expected to be replaced by AI, (2) functions still expected of human physicians, and (3) concerns about the medical field in the AI era. We also summarized the roles of physicians and patients, as well as the changes in the clinical environment in the age of AI. Some of the current functions of the physician were primarily replaced by AI functions, while others were inherited as the functions of the physician. In addition, “functions extended by AI” obtained by processing massive amounts of data will emerge, and a new role for physicians will be created to deal with them. Accordingly, the importance of physician functions, such as responsibility and commitment based on values, will increase, which will simultaneously increase the expectations of the patients that physicians will perform these functions.

**Conclusions:**

We presented our findings on how the medical processes of physicians and patients will change as AI technology is fully implemented. Promoting interdisciplinary discussions on how to overcome the challenges is essential, referring to the discussions being conducted in other fields.

## Introduction

Artificial intelligence (AI) is a term used to describe the use of computers and technology to emulate mechanisms of human intelligence, such as thought, deep learning, adaptation, and sensory understanding [1-3]. The AI revolution is said to be a new industrial revolution that will affect all industries, and health care is no exception [4]. In the field of clinical medicine, in particular, advancements in AI technology and machine learning, with their ability to constantly capture and optimize vast amounts of data, will have a significant impact on the accumulation and updating of medical knowledge, the discovery and acceleration of diagnostic methods [5], and the selection and implementation of treatment methods [6]. Thus, the impact of these technologies will force changes in all aspects of medical practice in the future [4,7-10].

Furthermore, the technological revolution and advancement of AI may cause implications for clinical practice, especially for physicians, allied health care professionals, and patients. Already, many patients are seeking advice on the internet before they see a doctor, which is influencing patient behavior [11]. Medical professionals are also said to be heavily influenced in their practice by the information derived from the internet [12]. These are already beginning to influence the decisions that patients and doctors make in everyday practice.

There are various opinions about the impact these AIs will have when used in society. Some of the positive effects include a more accurate selection of treatment methods and the reduction of the time burden of medical professionals. On the other hand, problems have been identified, such as selecting a diagnosis when the mechanical diagnosis differs from the medical one. This difference may be due to the technical limitations of AI, unethical decisions, and additional workload on the medical profession [5,13]. While many general practitioners in the United Kingdom are skeptical about AI implementation, they say that much of their work will be replaced by AI in the near future [14]. However, it is not fully clear how the information provided by AI will affect the patient-doctor relationship in actual practice.

Therefore, we conducted an exploratory study to determine how the introduction of AI services into the medical field will soon affect the role of the doctor or the doctor-patient relationship, how it will change the role of the physician, and concerns　associated with the change.

## Methods

### Study Design

We adopted a qualitative research design based on focus group interview methods.

### Recruitment Procedures of Participants

In this study, participants were recruited through snowball sampling from physicians residing in Tokyo’s suburbs. The investigators invited all participants through one of the author’s SNS with email to participate with predetermined inclusion criteria: medical professionals who currently work at least 20 hours a week in the medical field and who can give a basic explanation of the term “artificial intelligence.” We used convenient sampling to recruit participants but tried to include a diverse group in terms of age, gender, specialty, and workplace.

### Interview Procedure

The contents of planned interviews were explained to participants in writing in advance. Researchers conducted interviews at the date and time specified by the interviewees in an interview room in the Tokyo metropolitan area and ensured participant privacy. The interviews were conducted in line with the questions listed in the interview guide. We also provided 3 clinical scenarios about the decision-making of second opinions on breast cancer treatment, AI-automated remote self-diagnosis, and medical advice on health behavior based on AI-calculated risk calculation. We conducted 3 focus groups with a mix of physicians’ specialties, with 2-4 participants in each group. All interviews were led by a moderator and an assistant moderator (SB and MT), and all data were collected in January 2018.

### Analysis Methods

Interviews were recorded using an integrated chip recorder, and verbatim transcripts were created. Each sentence in the raw data was carefully read and analyzed. Two authors independently and repeatedly read through all the data and coded them according to meaning chunks. At the time of coding, no attempt was made to simplify expressions. Further, codes with similar content were grouped into a subcategory and given a name representing the shared content. They subsequently grouped similar codes into subcategories to provide insight into meaningful topics and codes. Finally, all results were merged and reconciled through repeated discussion among all authors. While creating subcategories, efforts were made to simplify their names so that the subcategory’s meaning could be readily understood from its name alone.

Further, similar subcategories were grouped into categories and then into core categories, with increasing levels of abstraction. The authors verified the content validity of the data and analyzed the reliability and validity by conducting discussions until they reached a consensus regarding classification, as well as coding. Data were analyzed using MAXQDA Plus12 (Release 12.2.1) software (VERBI GmbH). The interviews were repeated until data saturation was reached. Finally, the final results were prepared by asking the participants to review and comment on the summarized results and provide feedback.

### Ethics Approval

All the participants provided written informed consent and were assured that the contents of the interviews would be recorded and that their statements would be reported anonymously. All participants were compensated with US $71 prepaid card for the interviews, which lasted up to 2 hours. In addition, members of the research team took notes during the sessions to capture important elements. The ethics committee of the National Hospital Organization Tokyo Medical Center (Approval R18-134) reviewed and approved the study protocol. All methods were performed in accordance with the relevant guidelines and regulations.

## Results

### Overview

We found out the roles of physicians and patients, as well as the changes in the clinical environment in the age of AI through the focus group interview. Such views have not been found in the existing study. Some of the current functions of the physician were primarily replaced by AI functions, while others were inherited as the functions of the physician. In addition, “functions extended by AI” obtained by processing massive amounts of data will emerge, and a new role for physicians will be created to deal with them. Accordingly, the importance of physician functions, such as responsibility and commitment based on values, will increase, which will simultaneously increase the expectations of the patients that physicians will perform these functions.

The survey was conducted with 3 groups: A, B, and C. The same physician (SB) conducted the interview as the moderator every time. Each of the 3 groups recruited 4, 3, and 3 subjects. On the day of the interview, 1 participant in group C was unable to participate due to being unwell. In total, 9 participants (4, 3, and 2) in the 3 groups were interviewed. The group interview durations for the 3 groups were 103 minutes, 105 minutes, and 99 minutes, respectively. Content saturation and theme development were achieved with the 3 groups.

Three of the participants were in their 30s, 1 was in their 40s, and 5 were in their 50s. Seven were men and 2 were women. Four participants were general physicians or family doctors, 3 were psychiatrists, 1 was an emergency physician, and 1 was an anesthetist.

When analyzing the interview content, we deliberated on 3 core categories [Functions expected to be replaced by AI], [Functions that will still be expected of human doctors], and [Concerns about the medical field in the AI era] and were able to extract the axial codes associated with these categories. In the following descriptions, core categories will be expressed using [ ], categories using { }, subcategories using < >, codes using ‘ ’, and actual conversations using “ ”.

### [Functions Expected to be Replaced by AI] and [Functions That Will Still be Expected of Human Doctors]

For the medical process and the changes thereof after the implementation of AI, 3 categories and 5 subcategories (Table 1) were extracted regarding [Functions expected to be replaced by AI] within the scope of the current work of physicians. Moreover, 2 categories and 9 subcategories were extracted regarding [Functions that will still be expected of human doctors] (Table 2).

Some codes were extracted for [Functions expected to be replaced by AI], regarding the basic medical content of the current medical practice of physicians. Some representative ones among these are “AI can practice appropriate treatments widely,” and “AI can replace doctors in providing simple medical treatments.”

The following excerpts of conversations with 1 participant are representative statements regarding this theme.

Most likely, before such things emerge, simple procedures like influenza or common cold treatments will already be replaced. As a result, not that it will be a relief for doctors, it may be the case that the doctors that only provide such services will probably no longer be necessary. For certain, the work of physicians will change greatly.

Moreover, it has been pointed out that AI-based medical services will replace conventional medical services and have more advanced and enhanced functions compared to the current medical services provided by human physicians. Among these are {① subdivided algorithms~} and {② fact analysis~}. Both of these cannot be realized in medical services provided by humans and represent functions that are only possible because of AI.

In particular, opinions such as “AI can perform highly accurate diagnosis by consolidating information” and “AI can perform more accurate diagnoses” were obtained in relation to the <Clinical Reasoning> subcategory, which belongs to the **{**① subdivided algorithms~**}** category.

The following excerpts of conversations with 1 participant are representative utterings regarding this theme.

For example, for influenza treatment, say, the input related to personal symptoms, age, whether elderly people or children are living together, information about one’s workplace, etc., are completed, and then the button whether a quick kit test is required or not is selected. This will cause something to instruct the person to blow their nose and deposit the fluid into it. When this is registered, the computer will analyze and inform that ‘you are influenza-positive,’ which will be considered an influenza-positive result. But in addition to that, most likely, AI will also be able to calculate things like ‘found positive, but your probability of having influenza is about this much’.

Moreover, for <Information provision>, opinions such as “AI is better able to explain detailed information” were also expressed. These are opinions expressing thoughts that AI likely possesses the function to benefit patients by improving the accuracy of medical services provided.

**Table 1:** Core category: [Functions expected to be replaced by AI].

Category and subcategory		Code
**Replaceable functions in medical services**		
Basic patient care		AI^a^ can provide better medical services than doctors AI can replace doctors by providing simple medical treatments AI can practice appropriate treatments widely AI can replace pediatric treatments AI can perform more accurate diagnosis AI will provide appropriate treatments based on detailed information, making doctors and nurses redundant There will be no difference between the work performed by AI and that performed by doctors, nurses, and counselors
Communication		Listening and counseling about anxiety by AI will also be possible Communication will also be replaced by AI AI can also provide cognitive behavioral therapy AI may also be able to provide healing and energize patients AI is better at building rapport
**Functions extended by AI** **①** **~ Subdivided algorithms ~**		
	Clinical reasoning	AI can estimate treatment efficacy AI can consolidate information to improve diagnostic accuracy Computers will mediate between information and patient


	Information provision	AI is better able to explain detailed information AI can replace the work of explaining things to the patient

**Functions extended by AI** **②** **Fact analysis without cognitive bias and judgment (no bias cognition and judgment) (unbiased cognition and judgment)**		
	Excellence stability	AI can provide useful advice better than the physician who knows the patient through continuous treatment AI can provide treatments that are more standardized, less variant, and broader in range than those provided by doctors AI can respond to inquiries free of any conjecture (prejudice or bias) about other medical treatment providers Treatment outcomes may vary largely depending on the physician, but such a concern with AI is minimal




^a^AI: artificial intelligence.

**Table 2:** Core category [Functions that will still be expected of human doctors].

Category and subcategory			Code
**Partial replacement possible**			
	Clinical reasoning	AI^a^ presented a range of options with evidence will be limited	
	Information provision	Physicians can skillfully communicate information Unlike AI, physicians can tell detailed stories using colors and shades that AI does not possess	
	Excellence stability	AI cannot respond by considering subtle changes over time AI is not good at advancing treatment by taking into consideration the progress	
	Communication	It is difficult for AI to build a trusting relationship through emotional exchanges AI cannot intervene in doctor-patient relationship AI cannot match the power of medical services coordinated by various professionals AI lacks appropriate altruism	
**Difficult to replace**			
	Value-based Commitment	AI cannot provide personalized recommendation If the AI proposal differs from that of the physician, then the result should be consulted with a different physician AI may not be able to reflect its own values in providing explanations	
	Emotion sensing	Only the physician can listen and alleviate anxiety It is difficult for AI to deal with patients having suicidal ideation AI cannot adjust the degree of listening Physicians are more adept in providing healing and energizing patients	
	Sense sensing	AI is not good at taking into consideration general appearance AI is not good at medical treatment related to pain	
	Responsibility	The final decision cannot be delegated to AI Physicians have the role to approve AI proposals	
	VUCA^b^ agenda	The attitude of being concerned about complex problems will become important for physicians in providing medical services The physician must have the status of an advisor for the final decision It is a good idea that the physician takes the role of providing healing in the information society Physicians can perform the work that AI cannot perform Advice from the physician who knows the patient through continuous treatment will be useful in the AI era AI cannot provide medical services that have associated uncertainty	

^a^AI: artificial intelligence.

^b^VUCA: volatility・uncertainty・complexity・ambiguity.

Another category, {②Fact analysis without cognitive bias and judgment (no bias cognition and judgment) (unbiased cognition and judgment)}, concerns the improved continuous and fair medical services that AI can provide. This function has previously been performed by doctors. However, making it stable (continuous provision) and providing it fairly to every patient is possible only because of AI.

In particular, this is expressed by the content that “AI can provide useful advice better than the doctor that knows the patient through continuous treatment,” within the subcategory of <Excellence Stability>.

Moreover, for the treatment, the physician considers the information about the patient obtained through limited interactions in the clinic or clinical settings. However, in the case of AI, detailed information about past activities of daily living and a large amount of information that is difficult for humans to store and remember continuously can be accumulated and used. Accordingly, opinions were extracted, where the thought was that it would be possible to provide medical services that would have excellent stability (continuity) by taking into account all these information.

The following excerpts of conversations with 1 participant are representative utterings regarding this theme.

Assuming that the issue about whether or not we really know all about that person is still outstanding, not surprisingly, it may be that even if we are observing the patient over a long time, the patient may have intentionally not exhibited many things, which may be due to wavering of the mind depending on the situation the patient is in. It is likely that the patient only is capable of gently explaining various information from the internet even better by taking into account information about oneself.

Additionally, within <Excellence Stability> in the same category, “Treatment outcomes may vary largely depending on the physician, but such concern with AI is minimal,” was also found. The following describes the conversations with 1 participant in this regard.

For the operations performed by surgeons, I think the difference due to skills of the surgeons is more than that due to the data. The various consultations I provide are often about operations involving the waist or neck. Say, for waist surgery, based on published literature, I usually provide data to the patient showing the possibility of improvement, the possibility of no change in symptoms, and the possibility of worsening of symptoms. At the same time, I add the caveat explaining that these data are all compiled by surgeons and therefore who performs the operation is very important, and inform the patient that I can refer a surgeon if so desired. Beyond that point, I ask the patient to see the referred doctor and learn about the referred doctor’s success rate. I think such data only represent the average by taking an overall view. It is likely that this doctor only performs partial resections that result in better success rates. If the time comes, when the robot all by itself, depending on the definitive expansion to related stages, will perform the operation without any human intervention at all, it is likely that the diagnosis of such a computer system will be more reliable.

In addition, similar content like "AI can respond to inquiries free of any conjecture (bias or prejudice) about other medical treatment providers" was also found.

In <Excellence Stability>, 1 connotation is that while differences in treatment may result depending on the changes in the physical condition and mood of the physician, it is possible to avoid such pitfalls and provide a consistent and uniform medical treatment with AI. Moreover, there were opinions that it is possible to provide fair treatment without any bias with AI; a change in the mood of the physician owing to elements like patient status and treatment history could not affect the content of treatment.

In contrast, for the core category of [Functions that will still be expected of human doctors], 2 categories of {partial replacement possible} and {difficult to replace} were found, and for the {difficult to replace} category, 5 subcategories of <Value based Commitment>, <Emotional sensing>, <Sense sensing>, <Responsibility>, and <VUCA Agenda> were found (Table 2).

In particular, in <Value based Commitment>, there was the code of “If the AI proposal differs from that of the physician, then the result should be consulted with a different physician.”

With respect to this, the following describes the utterings of 1 participant.

In a situation where it is difficult to comprehend the detailed information obtained after consulting AI for diagnosis, realistically, the patient will likely go to another doctor with the information and go for a re-diagnosis. Kind of take this step before seeking a second opinion… However, depending on the temperament of the physician from whom the second opinion is sought, I think there may be physicians who are not convinced or, out of distrust with AI, may become angry. In the end, the situation will be a difficult one where the patient will have to choose from only two options, AI or the physician.

Opinions were also found in which participants believed that, while a diagnosis could be performed in this manner by aggregating detailed information, in practice, AI is unlikely to be able to personalize and share appropriate decisions that take into account the patient’s value perception and activities of daily living. Opinions stating that physicians who are human whose support will be required under such circumstances were also extracted.

**Figure 1:**
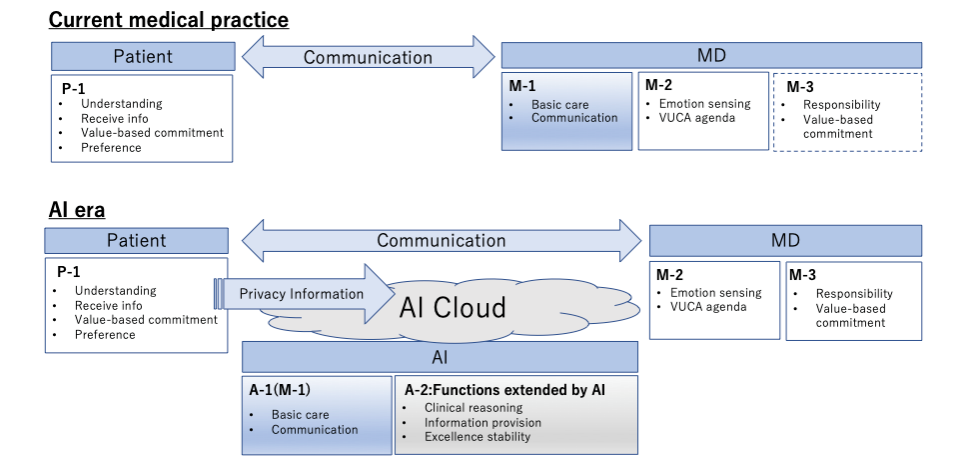
Roles of physicians in the artificial intelligence (AI) era. MD: Medical Doctor; VUCA: volatility・uncertainty・complexity・ambiguity.

Based on the above results, we illustrate in the following figure the roles of physicians and patients and the changes in a clinical setting in the AI era (Figure 1).

The upper part of Figure 1 shows the roles of physicians and patients in current medical practice. The roles of medical doctors were categorized into 3 types. The lower part shows that with the shift to the AI era, changes will occur with regard to the parties performing these roles and the related work effort. M-1 will largely be replaced by AI functionality, denoted by A-1 for the AI era. The M-2 and M-3 roles will continue to be medical doctors functions.

As for the information path, a vast number of patients’ personal information will be transferred from the patient to AI. AI will store and manage this massive number of patients’ personal information in the cloud, allowing it to provide detailed personalized information to both the physician and the patient. While a vast amount of information could be used this way, privacy protection has been pointed out as a potential concern.

### [Concerns About the Medical Field in the Age of AI]

In the interviews, the participants expressed concerns attributed to AI implementations. These concerns attributed to AI implementations were broadly divided into 3 groups (Table 3). One is the concern due to the functions extended by AI. It is about the physician’s confusion regarding dealing with the detailed information produced by AI. The following explains the concern in detail.

Firstly, <Understanding the AI thought process> includes “The reasoning process behind AI diagnosis is unclear” and “Physicians are fearful about not understanding the AI thought process.” In addition, opinions related to “AI with embedded-ethics will be required” in <Manipulating AI thought process> were also extracted.

One representative opinion in this regard is described below.

In the end, considering that humans are living beings, grey areas will always remain, and I am not sure when such a stage will come when it has to be decided whether human beings will ultimately provide judgment regarding these areas or forego such responsibility. Therefore, initially, it may be alright to leave it to the machine, thinking that humans may produce errors and the machine is always unbiased (innocent). But there is always a possibility that guided by such thoughts, humans will give up thinking by themselves altogether at some point, and when that happens, it may well be possible that the virtue of innocence (unbiased), which is taken for granted now, may not hold true anymore due to the unprecedented ways the system may learn and mature, and that is what concerns me.

This way, concerning the extended functions created by providing detailed information, the physicians expressed the difficulty of comprehending the information and their anxiety when confronted by such information.

Second, [Concern about Physician Potential Role] has also been expressed. As for <Responsibility>, there is concern about “Who will be responsible for the AI diagnosis?” Moreover, for <Value based Commitment> there is the concern that “Even adding AI support may not necessarily make it easier for the patient to make decisions.”

One representative opinion regarding this is described below.

I think the ultimate decision will always be made by the patient, and this decision-making will be supported (by AI). For example, let us consider the situation of a 45-year-old salesman who works full-time, has three children, and has a long life ahead. For such a patient, we may suggest if standard treatment and chemotherapy are undertaken, then the probability of cure is about this much, the n-year survival rate is this much, the burden of continuing treatment is this much, and the expenses will be this much. However, if the AI advice is followed, then most probably it is more definitive, but there will still be side-effects, and there will be things that should be considered afterward, and most likely, the patient will not be able to work for a few years. I think a decision has to be made by taking into account all such information and holistically judging the associated risks, merits, and demerits. However, it is doubtful whether the patient will be able to make a decision in such a situation.

**Table 3:** Core category [Concern about Medical Field in AI Era].

Category and subcategory			Code
**Concern about functions extended by AI^a^**			
	Understanding the AI thought process	Physicians are fearful about not understanding the AI thought process The reasoning process behind AI diagnosis is unclear It is probable that the assessment of the AI diagnosis by the AI developer may be different	
	Capability	As a physician, I am feeling fearful that AI can provide life-related important information Physicians who have not learned evidence cannot use evidence	
	Balance between physician and AI	Which one should be adopted, the physician’s diagnosis or the AI diagnosis	
	Manipulating AI thought process	It is difficult for AI to provide medical services compliant with ethical standards AI with embedded-ethics will be required	
**Concern about physician potential role**			
	Value-based commitment	Even adding AI support may not necessarily make it easier for the patient to make decisions I think allowing AI to make decisions regarding critical content will be a problem I think patients will be confused even if AI presents only detailed objective data	
	Responsibility	Who will make the decision and how will it be made Who will guarantee the quality of AI practice Who will have the responsibility for the AI diagnosis	
**Concern about patients**			
	Inequality	Whether AI medical services can be used or not will be determined by the financial affordability of the patient Whether good quality AI medical services can be used or not will be determined by the financial affordability of the patient With advance of AI into medical services, information about anxiety of patients will become widely known, which in turn will spur commercialization	
	Next action	There will be confusion about AI proposal depending on the patient	
	Privacy	There is a concern whether AI will be able to maintain the same level of tolerance as in the current medical practice for handling personal information There will be no privacy There is a concern about how privacy-based medical practice will develop sacrificing privacy for convenience	
	Experience	Patients will need to get used to AI medical services	

^a^AI: artificial intelligence.

## Discussion

### Principal Findings

This paper presents an exploratory qualitative study to determine how the introduction of AI services into the medical field will affect the medical process and the doctor-patient relationship. To the best of our knowledge, no previous study has deliberated with the due diligence of physicians on the introduction of AI services into the medical field. We believe we can ascribe a certain validity to our results based on in-depth deliberations conducted using group discussions involving currently active physicians. According to the results, some of the physician’s functions will be replaced by AI in the AI era. Moreover, some of the functions will be extended by AI, and at the same time, the potential functions that physicians have so far performed will also be extended more than ever before.

Assessments on the current competencies of physicians in various countries [15-19] have revealed that many of the desired competencies of physicians will likely be replaced by AI services. However, note that the results of our study are not the outcome of an attempt to find answers by directly asking whether the desired competencies of physicians as identified in various countries will be replaced by AI services. Accordingly, adequate caution is necessary when interpreting the results. Moreover, some physicians thought replacing doctors might be possible, while some physicians believed that replacing doctors in medical services would be impossible, indicating that there is no certainty. A similar opinion from the patient’s point of view has been reported in the past [20], and it is necessary to continue to investigate the substitution of AI for a part of doctor’s medical care. For example, both <Communication> and <Excellence Stability> are common subcategories extracted into 2 core categories. The present results are nothing more than answers obtained from currently active clinical physicians assuming a future scenario. Accordingly, it is necessary to keep monitoring how the AI services are implemented in clinical settings and what functions of the physicians are replaced accordingly.

Moreover, it is surmised that in the AI era, medical evidence will be even more personalized; it will be more detailed than current practice and have more specific individual information that will be shared with the doctor and the patient [21]. In this study, 1 finding is that “AI is better able to provide detailed information.” In other words, it can be said that such information could be provided free of any heuristics, a form of cognitive bias, that humans use in judgment or decision-making in the field of decision science. Realistically, humans often make decisions that are not rational, many of which are considered direct results of applying heuristic bias [22]. Whether irrational decisions result in wrong results is beyond the scope of discussions here. However, it can be expected that such tasks for physicians are reduced and that the time saved due to such reduction will be used for other tasks.

Next, for the functions still performed by physicians, value-based commitment and emotional sensing were identified. This is most likely because the physician will be more capable than AI in rationing the patient’s values and preferences, which are important in decision-making. There may be other geographical regions with a prevalent culture where the preferences and values of patients are not appropriately shared among all parties concerned, as in Japan [23]. In such cases, these need to be adequately clarified before making decisions. Two reasons have been identified as to why human physicians are considered better at grasping the patient’s preferences and values even in the AI era. One is that it is very difficult for AI to grasp things that are not quantifiable. Preferences and values are things that cannot be quantitatively measured using a scale of 0-1. Even though AI is very competent in accumulating quantified data, quantifying technology is presumed to be difficult. Our findings suggest that these functions will continue to fall under the purview of human physicians because understanding the patient’s preferences and values involves multiple factors and elements, but this requires further consideration.

The other is that it is perceived as difficult for AI to comprehend context [24]. It is likely possible for AI to extract quantified preferences and values [25], but the context is distinctly different before and after a preference or value is born. That is, it is difficult to understand what process resulted in the preferences and values that have been quantified [26,27]. Assuming that such a division exists, it is difficult to say that applying quantitative preferences and values in decision-making is appropriate. Based on these reasons, it is believed that it will be difficult for AI to handle patients’ preferences and values comprehensively.

Lastly, we discuss the desired roles and competencies of physicians necessary in decision-making in the medical field in the AI era.

It is believed that {Functions that will still be expected of human doctors} will constitute the core of the roles performed by physicians in the AI era. However, based on the {Concern about the Physician’s Potential Role} found in {Concern about the medical field in the AI era}, we believe the following 2 elements will be important in exploring the desired model physician in the AI era. One is the determination of commitment as a professional in decision-making. The other is the capability to support personalized decisions based on detailed medical information, that is, excellent consulting capability.

As the results of the study indicate, because the vast amount of detailed information produced by AI results in increased burden and difficulty in interpreting such information, there is a concern that the physician may forego their responsibility concerning such information and the decision thereof. Under such conditions, the physician needs to possess the determination of commitment and support decision-making without evading their professional responsibility while promoting mutual communication with the patient. That is what we believe is the desired responsibility of the physician hereafter. We have identified this as one of the important competencies of a physician in the AI era, “Responsibility of decision-making.”

Moreover, excellent consulting capability as a decision-making professional will likely become essential. As explained earlier, before even considering detailed information, it is difficult for AI to consider patients’ values, wishes, and surrounding conditions because they are difficult to quantify. Especially, commitment in decision-making must be realized by focusing on the elements that cannot be quantified, spending effort to grasp the narrative up to the point such elements are created, and learning ways to integrate unquantifiable elements in multidimensional ways. We believe that the maturity of such functions and the growth of technology will constitute the challenges that physicians should actively engage in, in the AI era.

### Study Limitations

The following describes the limitations of the study. First, the sample size is small, limiting the study’s generalizability. The results were derived from opinions of 9 physicians only, and there were wide variations in men to women ratio, age, and years of experience. Furthermore, there were fewer surgeons among the participants and more internal medicine or family medicine doctors, indicating a bias in a medical specialty. There are many other medical professionals in clinical settings, such as nurses, pharmacists, occupational therapists, and physiotherapists, who have naturally different viewpoints. Moreover, concerns from the viewpoint of doctors may be very different from the concerns of a patient.

The second is about the inadequate common perception of AI among the participants. Although careful consideration was given to stimulate discussions in the interviews and interview guides were used to the extent possible to teach a common perception of AI, we note that there were limitations in this regard.

Lastly, to improve the trustworthiness of the analysis results, a member check was conducted after the data content saturation was confirmed through repeated discussions among the authors. However, we believe that trustworthiness can be further improved through triangulation, such as by combining our analysis with quantitative studies.

### Conclusions

We presented our findings on how the medical process of doctors and patients will change after the implementation of AI technology. Research has just begun on how changes are expected to occur after the introduction of such technology and how physicians should respond to these changes. It is necessary to promote further research and, at the same time, to promote interdisciplinary discussions on how to overcome the challenges, referring to the discussions being conducted in other fields.

## Abbreviations

AI: artificial intelligence

## Data Availability

The data that support the findings of this study are available from the corresponding author, MT, upon reasonable request.
